# The emerging institutionalisation of knowledge co-production in sustainability research

**DOI:** 10.1007/s13280-025-02161-5

**Published:** 2025-03-18

**Authors:** Janina Käyhkö, Mikael Hildén, Ia Hyttinen, Kaisa Korhonen-Kurki

**Affiliations:** 1 Ecosystems and Environment Research Program, University of Helsinki, Helsinki, Finland; 2 The Finnish Environment Institute (Syke), Helsinki, Finland

**Keywords:** Knowledge co-production, Knowledge user, National level, Sensemaking, Science-policy interface, Strategic research

## Abstract

Co-production of knowledge in science-policy interfaces has received increased attention as a way of addressing grand societal challenges with the hope that it will create a base for enacting transformative change. We explore the development and outputs of projects funded by the Strategic Research Council of Finland, which has had the aim of generating strategic knowledge through interactions with stakeholders. We examine how producers and users of knowledge understand co-production as revealed by a researcher survey and stakeholder interviews in relation to different domains in the institutionalisation process. Our results show advanced, emerging and explorative levels of institutionalisation of knowledge co-production practices and highlight the differences between needs-based and transformative approaches to co-production. We conclude that to succeed, efforts to institutionalise knowledge co-production should recognise several societal domains from governance to resources and culture. Advanced institutionalisation for the co-production of transformative knowledge can significantly strengthen the potential of sustainability research.

**Supplementary Information:**

The online version contains supplementary material available at 10.1007/s13280-025-02161-5.

## Introduction

Complex interrelated societal and environmental challenges, such as climate change, the loss of biodiversity and the overexploitation of natural resources, demand new ways of producing, interpreting and applying knowledge (Funtowicz and Ravetz 1993). Transdisciplinary research^1^ aims to address such societal challenges by using participatory approaches to create solution-oriented, socially robust and transferable knowledge for both scientific and societal practice (Pohl and Hirsch Hadorn 2007; Lang et al. 2012). New ways of producing knowledge are expected to contribute to transformative societal change. Changes in the production of knowledge and solutions to societal problems affect science-policy interfaces (SPIs) and the division of responsibilities (Maas et al. 2022) as well as the dynamics of such key components as trust and values (Hurlbert and Gupta 2015; Jagannathan et al. 2023).

The co-production of knowledge, in which researchers collaborate with stakeholders to generate new knowledge, is assumed to enhance the societal impact of research. Scholars have argued that sustainability challenges can best be addressed through an iterative process directed at societal change that integrates different ways of knowing and acting (Wyborn et al. 2019). High-quality knowledge co-production in sustainability research is often assumed to be local or case specific (Norström et al. 2020). However, a case-by-case approach may fail to develop a general and strong base for broader science-policy interaction. An institutionalisation of knowledge co-production (e.g. van der Hel 2016; Owen et al. 2021) may alleviate these risks. Institutionalisation has been described as the process by which social processes, obligations or actualities come to take on rule-like status in social thought and action (Cooper et al. 2008). Scott (2014) has argued that institutionalisation is based on regulative, normative and cultural-cognitive elements which establish a new societal practice. In the case of knowledge co-production the institutionalisation would ideally support wide application and encourage science-policy interaction that ultimately increases the societal impact of co-created knowledge. However, the empirical research on the process and impacts of the institutionalisation of co-production of knowledge is still fragmented and inconclusive. The focus has been on specific approaches or concepts (e.g. Cairns and Krzywoszynska 2016; Fasina et al. 2021) as well as programmes and projects (e.g. van der Hel 2016), but the views of stakeholders, which are critical for commitment, resources and the emergence of a new culture, among others, have seldom been examined.

The ambitions in projects that researchers label as engaging in co-creative science-policy interaction may range from relatively practical problem solving to the pursuing of deeper transformative change for sustainability. This scale in ambitions may be explained by differences in the epistemological and ontological assumptions and choices in the research design. However, it is seldom explained clearly to the participating stakeholders. The stakeholders may, depending on the project, be invited to truly co-produce new knowledge, become selected targets in dissemination efforts or just function as tokens for fulfilling the funders requirements. We argue that further institutionalisation of co-production in science-policy interfaces can develop in many ways but to understand the opportunities and challenges of institutionalisation it is relevant to explore how researchers from different disciplinary backgrounds approach knowledge co-production, and how the stakeholders in the policy-sphere perceive interactions labelled as co-production.

Research funded by the Finnish Strategic Research Council (SRC) provides an interesting study case and a source of insights on co-production of knowledge because the SRC has had an explicit goal to foster new and deeper types of science-policy interaction. The creation of the Strategic Research Council (SRC) in 2015 was an innovative decision to fund research with the explicit aim of generating strategic knowledge through interaction with stakeholders. It represented a first step in the institutionalisation of knowledge co-production by providing competitive research funding that supports new types of science-policy interactions. A recent study and an evaluation of the SRC have shown, for example, that engagement of policymakers and researchers in knowledge co-production has been successful in the different phases of most projects, but deeper integration particularly in the latter phases of the projects and the research-based policymaking is not yet widely adopted (Kivistö et al. 2022; Korhonen-Kurki et al. 2022). There have also been other efforts to enable co-production practices in Finland, notably by establishing the Government’s analysis, assessment and research activities (VNTEAS), which was created as a parallel funding instrument simultaneously with the SRC to initiate short-term one-to-two-year long projects directly serving policy development.

In this paper, we analyse the development and outputs of co-creation in projects funded by SRC to support sustainability transformations. We focus on both the producers and users of co-produced knowledge. Our main research questions are:

1. How has knowledge co-production been understood and implemented in research projects funded through an instrument that specifically emphasises science-policy interaction both as an approach of doing research and as an outcome of the projects?
2. What levels of institutionalisation have been achieved in the co-production and what opportunities and challenges have been encountered?

To answer these questions, we apply a content analysis to understand how knowledge co-production has been perceived to influence research and policy making. By examining different aspects of institutionalisation, we explore the opportunities and challenges that have arisen and discuss how they may affect further institutionalising of knowledge co-production beyond the research funding. We also briefly reflect on other efforts to create novel science-policy interfaces. Based on this, we discuss lessons learned about shared challenges in science-policy interfaces and what could motivate Finland and other countries to institutionalise knowledge co-production to support research-based policy development for sustainability transitions/ transformations.

## Key concepts and analytical framework

### Knowledge co-production in sustainability research

Sustainability research that seeks to address increasingly complex, urgent global challenges has been a key area in the operationalisation of knowledge co-production (Miller and Wyborn 2020; Norström et al. 2020). Sustainability research stresses interactions between different forms of knowledge. This underlines an instrumental use of knowledge co-production (Apetrei et al. 2021), although co-production can also be seen as process for democracy and “deeper” (Mode 1) transdisciplinarity (Rigolot 2020). Distinctions can also be made at the level of participation of the different actors in the co-production process (Hurlbert and Gupta 2015) ranging from limited consultation in developing background knowledge and insights of a problem, to proposing actual solutions and taking responsibility for implementing them.

Chambers et al. (2021), focusing ultimately on co-producing concrete solutions to societal problems, have suggested ways of assessing how four basic themes are dealt with in the co-production process. First, the overall purpose of the co-production process may focus on either solving or reframing a problem. Second, the view on agency affects whether the process seeks to directly change the behaviour of actors or strive to shape systemic agency. Third, the recognition of political power may lead to attempt to influence those in power or seek to empower marginalised groups. Fourth, pathways by which impact is sought may focus primarily on transferring scientific knowledge or on creating new relationships between the actors involved. All of these are relevant for our case, in which the government has decided to create a funding instrument for research that should help in addressing major societal challenges.

The view of what co-production is or should be also affects how it is put into effect. We refer to the sensemaking of the key actors, in our case, researchers and stakeholders, as a process of creating an understanding of knowledge co-production and applying it in action (*cf*. Wibeck and Linnér, 2021). A starting point here is that different understandings of knowledge co-production are likely to shape the institutionalisation (van der Hel 2016) by affecting, for example, what kind of governance should be developed and what standards and routines should be established.

### Institutionalisation

The adoption of knowledge co-production in society as a practice goes beyond single researcher-driven initiatives that are employed from time to time in research projects. It is linked to the emergence of broader science-policy interfaces (SPI), which also can be seen as fora for mutual learning (e.g*.* Hurlbert and Gupta 2015) and knowledge governance (Van Kerkhoff and Pilbeam 2017). A society-wide adoption of knowledge co-production requires processes of institutionalisation. Building on Scott’s (2014) three elements in institutionalisation, Kuchenmüller et al. (2022) have conducted an extensive systematic review and identified six key domains that are relevant for the institutionalisation of evidence-informed policy making. We argue that these domains can also be applied in examining the institutionalisation of knowledge co-production. They are (1) governance; (2) standards and routinised processes; (3) partnership, collective action and support; (4) leadership and commitment; (5) resources; and (6) culture (see Kuchenmüller et al. 2022, p. 7-8). The domains cover both rules, power and values that are important in examining institutionalisation (Cooper et al. 2008). For example, changes in governance are needed to establish societal or organisational rules that secure SPIs and idea-led changes that establish transdisciplinarity as a shared value in society (*cf*. Max Weber, see, for example, Beckermann 1985). Partnerships ensure continued engagement in joint problem solving. This links with the suggestion of Joshi and Moore (2004, 40), who argue that institutionalised co-production is “the provision of public services (broadly defined, to include regulation) through regular, long-term relationships between state agencies and organised groups of citizens, where both make substantial resource contributions”.

We apply the domains of institutionalisation with a specific focus on the co-production of knowledge and the contribution of organised groups of researchers (in the form of funded projects) and policymakers from the public and private sphere. This goes beyond an institutionalisation that underpins the development of regulatory science, which, for example, Jasanoff (1995) has explored in depth. We argue that the institutionalisation of knowledge co-production proceeds gradually to different levels in the domains of institutionalisation, partly depending on how it is understood and conceptualised.

### Analytical framework

**Fig. 1:**
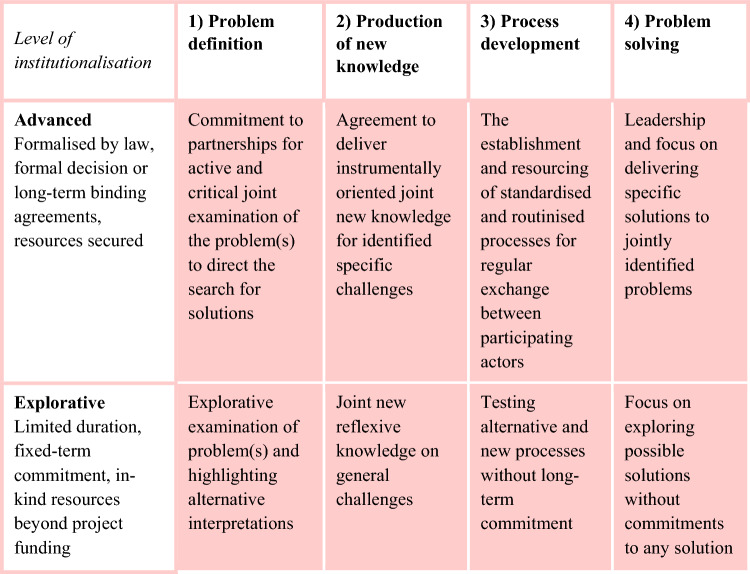
Analytical framework integrates the knowledge co-production practices and their level of institutionalisation

We examined knowledge co-production in the projects funded by the Strategic Research Council (SRC) by analysing practices in the four elements derived from Chambers et al. (2021) and linking them to different levels of institutionalisation (Fig. 1). The delivery of new co-produced knowledge (column 2) and the process development (column 3) focus on practical actions that aim at defining and solving the problems at hand. In the framework, we distinguished between advanced and exploratory institutionalisation. Advanced institutionalisation should qualitatively demonstrate the existence of established practices based on structures such as a working group that have been created for regular interaction between the parties involved in the co-production process. There should be key elements of the co-production process indicating routines, commitment and partnerships. Exploratory institutionalisation includes precursors to potentially more lasting solutions. It includes ad hoc contacts and testing elements of co-production but lack, among other things, formal agreements or long-term plans and commitment for the co-production. Operationalisation of the framework is described in more detail in Supplementary Information S3.

## Materials and methods

We explored science-policy interaction and co-production of knowledge under the Strategic Research Council (SRC) that from its initiation aimed at bringing in new elements in the SPI (see Supplementary Information S5 case description). We analysed the various forms that this institutionalisation of knowledge co-production has taken and the practices that have emerged and changed as a result.

The dataset was derived from an online survey of 25 SRC-funded research projects dealing with different aspects of sustainability transitions, and it covers more than half of all the projects that had been funded by time of data collection. The survey covered the methods and depth of integration of the research and interaction activities as well as the background, processes and challenges informing the knowledge co-production activities (Table 1).

**Table 1:** Survey information

Respondents (knowledge producers)	n
Consortium leader (project leader)	9
Coordinator	1
Societal interaction expert	8
Principal investigator	11
Researcher	9
Total	*38 (from 25 different consortia)*

Survey: The survey consisted of open-ended, multiple choice and Likert-scale questions that covered wider scope than that of the study at hand. For this study, the open-ended and Likert-scale questions on the themes of knowledge co-production practices and perceptions on knowledge co-production process were relevant and analysed

Sampling: The survey was circulated via e-mail in November 2020 to all consortium leaders, coordinators and societal interaction experts involved in ongoing current and recently completed projects, with a request to forward the survey to the principal investigators and other key persons

Analysis: The answers to Likert-scale questions were quantitatively analysed using Microsoft Excel for descriptive statistics of the respondent totals (Chromy and Abeyasekera 2005). Open-ended responses were subjected to a qualitative analysis (described below). The sampling unit is the project; hence, respondent means were analysed for projects that received more than one response (Chromy and Abeyasekera 2005)

In addition, 16 expert interviews were arranged online after the survey material had been analysed for the knowledge users’ perceptions of knowledge co-production and how they reflect those of the knowledge providers. The interviews were semi-structured, which allowed the discussion to flow between the topics while still maintaining a set order with respect to the perspectives of the interviewees during the discussions (see Supplementary Information S1).

Interviewees included knowledge users involved in policy making or enablers of the institutionalisation of knowledge co-production who were familiar with the process and outputs of one or more SRC projects. The participants (see Supplementary Information S2) represented three key stakeholder groups identified based on the survey, i.e. persons involved in national-level policy making (ministries), regional/ municipal policy making and strategically important industries. We ruled out participants from the current SRC organisation to avoid potential bias, while including some interviewees with a background in developing the SRC and the organisation since their viewpoints were considered valuable for understanding the basic aims of the funding instrument.

The survey and transcribed interview material were subjected to a qualitative content analysis by using our analytical framework (Fig. 1) and comparing the answers for similarities and differences. A quantitative analysis of selected survey material (see Supplementary Information S4) complemented the qualitative analysis. The framework was operationalised into a coding matrix (see Supplementary Information S3) for categorising the quotes from the interview material and the open-ended responses from the survey. The analysis was carried out iteratively by examining first the a priori codes and categories and identifying empirical codes (Gibson and Brown 2009) and then re-examining the material until all relevant quotations were identified and categorised coherently and a comprehensive understanding of the sensemaking was formulated.

## Results

### The purpose and practices of knowledge co-production in SRC projects

**Fig. 2:**
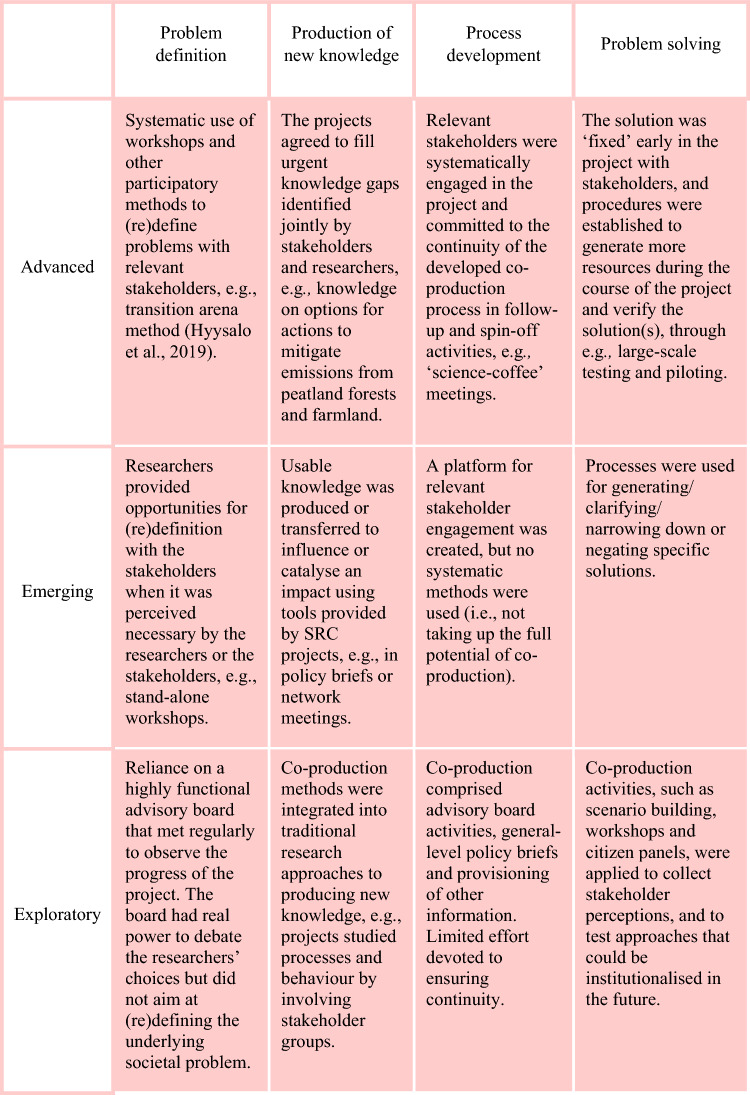
Framework together with illustrative examples from the collected data

The SRC projects fulfilled and developed elements of knowledge co-production sensemaking in different ways. Thus, they reflect different levels of institutionalisation of knowledge co-production. Based on the results, we supplemented the original framework by a category of “emerging institutionalisation” that went beyond the mere exploratory activity but could not yet be classified as advanced. In all four elements of co-production from problem definition to problem solving, we observed this range of institutionalisation (Fig. 2).

In problem definition, some projects achieved rule-based practices that can be replicated, whereas others displayed emerging institutionalisation in the creation of systematised dialogues (Fig. 2, “Problem definition” column). The exploratory institutionalisation was seen in projects that studied how stakeholders redefine problems, without intention to institutionalise the practices. Overall, the projects developed partnerships in problem definition, which can be seen to create the base for future collective action.

The production of new knowledge in interaction with stakeholders had been a starting point for all SRC projects, but the level of institutionalisation achieved or sought differed (Fig. 2, “Production of new knowledge” column). In general, the knowledge co-production practices became more standardised and routinised as the activities progressed. However, most remained at an exploratory or emerging level without firm structures or established practices that would have ensured long-term commitment to co-production beyond the lifespan of the project. Processes of gaining a shared understanding with the stakeholders of proposed solutions were first step towards the institutionalising of the knowledge co-production process but was not sufficient for achieving standardised and routinised practices during the lifetime of the project. This suggested insufficient commitment beyond the duration of the project and may also reflect lack of full appreciation and leadership to strengthen institutionalisation.

Process development for knowledge co-production was guided by the SRC funding criteria that required each project to, for example, appoint an interaction coordinator. The degree of standardisation and routinisation and the nature of the practices in the knowledge co-production processes were affected by the types of projects and the societal position of the principal investigators and research groups. Some projects were from the outset integrated in the institutional base for future activities at various scales. Projects that initiated stakeholder interaction to achieve the project’s research goals could be seen to explore the base for future institutionalisation (Fig. 2, “Process development” column).

The societal challenges that SRC projects addressed required multiple solutions. Some parts of the co-production of solutions could be institutionalised, while other parts remained exploratory. There were projects that specifically sought to introduce novel tools for finding solutions, such as citizen panels (Fig. 2, “Problem solving” column). These could be classified explorations of institutions for problem solving, whereas other projects were advanced in institutionalising the solving of specific problems.

### The two ways of making sense of knowledge co-production

We identified two dominant ways of making sense of knowledge co-production in the knowledge co-production processes of the SRC projects and the views on the institutionalisation of knowledge co-production practices. A needs-based sensemaking perceives co-production as a way to gain specific instrumental knowledge for dealing with issues constituting societal challenges. A transformative sensemaking emphasises a wider understanding of the challenges and sees co-production as a way to produce knowledge for the transformation of society.

#### Research for solutions to societal problems—needs-based sensemaking

Many interviewees saw that key aims of SRC projects should be to deliver new knowledge about complex and urgent societal challenges and to develop management or governance of solutions. Such instrumental aims can be seen as the starting point of the SRC themes. The interviewees generally recognised the need for transdisciplinary problem-solving skills. However, several interviewees and a few survey respondents criticised SRC projects for not being effective enough in addressing the problems. This criticism reflected a needs-based sensemaking that sees knowledge production as a service to policy makers and society. In the interviews, respondents sometimes portrayed the integrity of research almost as a nuisance, and projects were criticised for the lack of concrete outputs and for requiring stakeholders to devote too much time to participation in co-production activities. For instance, several respondents did not accept that co-production would require also stakeholders to learn to use new concepts or that far-reaching scenarios are needed to examine potential responses tackling the societal challenges. The opportunities for an open dialogue with stakeholders to build a common understanding of problems were rather seen from an opportunistic perspective, i.e. becoming relevant if useful perspectives were assumed to emerge.

However, several interviewees mentioned that the structures created to support the co-production and societal impact of projects have proven valuable and necessary for successful knowledge co-production. Such views encouraged institutionalisation through routinisation of practices. For instance, most interviewees had either been involved in or given the opportunity to take part in identifying the knowledge needs of the stakeholders during the project, and some had been involved already in the planning phase of projects. One ministry-level stakeholder noted that the SRC projects are generally perceived to be a beneficial source of learning and they aim to support further collaboration:

[In our role we] have always tried with our internal expert team to find the best possible officials in the most relevant field possible for each of these SRC projects, and then arrange a meeting between the researchers and these officials. They have been very good meetings. There is certainly some progress in them, so hopefully progress has been made, instead of passive board meetings on the part of our participants. [---] They are multi-year projects, they provide an opportunity to be in constant contact with researchers and train oneself to better understand the world from the perspective of researched knowledge, and also to inform the researchers about the boundary conditions for policy preparation, on what kind of information could be used. Not to steer that research in a certain direction, but so that researchers know that when there is leeway for action, it can be seen what can be done together. (Interview 9)

The survey results revealed that some researchers/knowledge providers saw their projects primarily in a needs-based light. This approach was prominent among project leaders who had a background in applied fields of study, such as business studies or agriculture and forestry research. These projects were primarily motivated by stakeholder-informed knowledge gaps and a need for practical solutions or business opportunities linked to broader societal challenges. One forestry researcher highlighted that they prefer the term co-research rather than co-production of knowledge for their project, which emphasises less the role of new knowledge while stressing the need to find solutions. The respondent referred to the tradition (culture) in the field:

Researching issues together and taking the research findings into practice together with the knowledge users has been customary already previously in forestry research. However, perhaps this type of approach [co-production] has become more popular, too. (Survey response)

The results of the survey and the interviews revealed differences between researchers and stakeholders in terms of the intended outcome and implementation of the research. While more than 80% of the survey responses (see Supplementary Information S4) suggested that knowledge co-production with stakeholders had affected the direction or execution of the research in the project, the interviewees saw mainly minor changes in how the research was conducted. These interviewees had noted only relatively small adjustments in the projects as illustrated in the below quotes from participants that criticised, first, the project and second, themselves for lack of what they perceive as sufficient communication between the “two sides”.

[I]f once a year we actually have this, what is called as a steering group [---] then [for] keeping track of what is going on [in the project] and providing comments, I mean, it could be more often. I understand that there needs to be academic freedom for researchers and the space for them to work and we are not expected to validate the analysis or results, but perhaps the project has just remained [---] a bit distant for me. (Interview 4)

Perhaps there is also need for self-criticism, as we sit in a lot of project boards, that we would also be more active in contacting the researchers and ask: “could you now come and tell us [about the research]?”. And just call our personnel in food department [to come and listen]. And usually the people have participated [in similar events]. But I think it might also be our task, who sit in those boards, to bring that knowledge into our workplaces. (Interview 2)

Regardless of the seemingly minor adjustments in the understanding of the problem or research practices through needs-based knowledge co-production, the outcomes may be significant. An illustrative example, confirmed by both stakeholders and researchers, is one project in which an additional scenario on restoration had been introduced as a peatland management option in the early phase of the project by a well-argued suggestion from the advisory board and which became highly policy-relevant later on. The limited stakeholder participation in needs-based approach was also justified with a focus on addressing a pre-identified and broadly agreed knowledge gap that had prevented the introduction of the proposed solutions. For example, researchers and stakeholders broadly agreed on the need to regularly co-produce knowledge on peatland forest and farmland management options that would have an impact on the climate policy in the land use, land-use change and forestry (LULUCF) sector.

Finally, the needs-based approach was also reflected in projects where an institutional base for co-production did not yet exist, and the co-production of new knowledge resembled a research method. In this aspect of the needs-based approach, the knowledge co-production methods were seen to be necessary for meeting project aims, but the use of co-produced knowledge was left to societal stakeholders to consider. For example, a project that focused on socio-environmental disruptions aimed to develop tools for handling unprecedented situations but did not have plans or ambitions to implement the tools through co-production or its institutionalisation. The key methods were described as simulation exercises for managing unpredictable changes that: “involve close interaction between the experts and the decision-makers during the planning, implementation and evaluation” (Survey response). A stakeholder discussed the relevance of the process and the importance of putting the learnings into practice referring to actions needed on their side as follows:

[This project], it sort of opened a view to the kind of scenario working and causal relations that is very hard to put into some sort of set of rules. [---] And the message from there [the study] was quite strong in that we need to tune our own systems in a way that acknowledges unpredictable changes there. And it is currently an open question if we can even have that type of set of rules. We have now acknowledged the issue, and now we need to figure out if it is actually this group [a municipal department] that identifies those signals and presents them to the decision-makers, to then further develop those rules, guidelines. (Interview 3)

Furthermore, the following quote illustrates how co-production practice is understood as “throwing the ball” from the research projects to the decision-makers.

[The SRC project works] sort of like a megaphone in the sense that it gives the research and its findings more space in the public discussion, with the means we have in use. A lot of relevant people are reached, and it is, in a way, mutual problem solving since it is based on research-based knowledge but also requires that someone hears it and acts upon it. (Interview 11)

#### Creating platform for open dialogues—transformative sensemaking

The interviews that reflected a transformative approach looked at the structures where societal problems arise instead of focusing solely on a specific problem. Environmental or natural resource governance was raised as examples of highly institutionalised practices that could be changed with more citizen participation. One ministry-level stakeholder highlighted this:

At least all these democracy projects that have developed co-design or citizen deliberation, these are good examples, in my opinion, of what could find their way into the government structures in long term and enable stronger agency [for citizens and other co-design participants] in natural resource and environmental issues, as well as [in] all youth related issues. (Interview 14)

The interviewees who emphasised a transformative approach felt that institutional structures for knowledge co-production were necessary steps towards change in the science-policy interfaces. They claimed that the current interaction between researchers and policymakers is not suitable for dealing with wicked societal problems and does not support researchers and policymakers to find new ways to approach them. The SRC projects were often seen as a new way of doing that type of research that they had been missing:

In a way, [the multidisciplinary view would be missing] if there were no researchers who would provide us with different perspectives on, say, the different security and human rights aspects of electrification and the sufficiency of natural resources [---]. Even if emissions are solved, many other problems can be created at the same time. It is the strength of these types of multidisciplinary projects that they are able to bring all this [together], in a way, they can bring the efficiency aspect of solutions, the fairness aspect and, in a good case, how it fits into our governance system, into the legislative system, so that they do not create solutions that, for instance, cannot be implanted in it. (Interview 6)

And, when they [industry participants in co-production], maybe look at things mainly from just the perspective of their own industry, and yet the energy sector here has a lot of social implications as a whole anyway. It was opened up really well and that's why we had a good discussion overall. [---] I also gained knowledge, the knowledge added a bit of pain, but this is the better way, and maybe now it is also possible to emphasize and clarify one’s own viewpoint so that it does not narrow too much to a certain area of expertise, but that one knows a bit broader, overall. (Interview 15)

These interviewees argued that both the integrity of research and the efforts to build trust between researchers and stakeholders are highly valuable. However, they expressed a concern that policymakers may sometimes be too strongly involved causing potential bias. Thus, partnerships can be a double-edged sword in institutionalisation. The concern is related to the division between the needs-based and transformative approach to knowledge co-production in general, which is illustrated in the following by a ministry-level stakeholder:

[F]rom the point of view of nature conservation, for example, it is good to always consider what does the knowledge user, if such a term is used, what exactly do they want from cooperation with strategic research? [---] [they often] want stronger arguments for their own opinions that have already been formed. It is perhaps quite rare to simply want to create new knowledge in a sincerely neutral manner and then form a description of the situation together based on it. This is also happening, and it could happen more. And here SRC could be one good instrument to advance this cause, this kind of reflection and creating a situation description together. (Interview 13)

The empowerment of marginalised groups was a key goal for some of the projects. These built on empowerment-focused research approaches, such as action research and citizen engagement, and focused on developing and applying societal interventions, such as democracy experiments, citizen panels and participatory approaches that activate the “unusual suspects”. One interviewee describes such process as follows:

[T]he starting point was how to consult young people so that participation does not accumulate [for an already active group in sustainability themes], and then we carried out digital consultations with the project in collaboration with schools with groups that certainly would not have participated otherwise. (Interview 6)

In addition, the advisory boards for the SRC projects were often perceived as having a greater role in empowerment efforts than usually in research projects. The advisory boards gave voice to stakeholders such as environmental organisations and other third-sector actors, which have often been marginalised in policy development. One interviewee had a relatively utilitarian view of interaction that involves empowerment aspects and highlighted the importance of networking as a resource:

I thought that yes, one of the key experiences, having worked for an environmental organization for seven years, for me is that there are environmental organizations that have relatively little social power or resources. And the way these existing projects probably strengthens this is in terms of knowledge that they create and that organizations can use [as a resource]. And they create relationships that are important in the activity of interacting with the researchers who are in that field, and both of these are somehow realized. (Interview 10)

Researchers also brought up methodological challenges and gaps that had arisen in transformative co-production projects on sensitive research topics with high societal relevance such as land use with multiple colliding interests or topics related to national security and preparedness for emergencies. Co-production of knowledge in ongoing conflictual political situations or in the development of deliberative societal decision-making demands special skills to avoid deadlocks. The institutionalisation domain of governance is critical in this respect. Furthermore, some interviewees noted that while it is desirable to engage citizens in the co-production of knowledge with high societal relevance, it is often very challenging to achieve it in practice.

The transformative approach of knowledge co-production differed from the needs-based approach of knowledge co-production in terms of how interviewees perceived the outputs and success of the projects. The transformative approach of knowledge co-production recognised that the relatively high operational costs or inputs of the projects were an inevitable part of the changes and accepted that the outputs may not be concrete or measurable during the lifetime of the project but are instead likely to evolve over time. However, there were promising examples of initial impacts, of which the transition arena method process provides an illustrative example. The transition arenas involved a group of experts, including stakeholders, who gathered for a lengthy, intensive process lasting 3–4 months to gain a shared understanding of the problem and the transition opportunities. Some projects were able to feed into or modify existing structures so that they became oriented towards co-production. Examples included regular meetings with civil servants to discuss and interpret research findings. For instance, the Ministry of the Environment has regularly invited project members to interact with ongoing policy processes, such as work to develop a circular economy. Some of these practices have become routinised and were considered likely to continue.

## Discussion

### The diversification of knowledge co-production in sustainability research

The literature on co-production (and co-creation) for sustainability transitions and transformations has grown considerably (Bremer and Meisch 2017; Chambers et al. 2021; Lukkarinen et al. 2023). At the same time, the views on what co-production entails have become more diverse (see, for example, Bremer and Meisch 2017; Chambers et al. 2021). Our study has highlighted diversity in applications and interpretation of co-production. By collecting primary data from both researchers and stakeholders, we identified two dominant ways of making sense of knowledge co-production that are relevant also for a general discussion on science-policy interfaces (SPIs): the instrumental response to the needs of public bodies and other stakeholders for solving practical problems vs. new ways of producing knowledge for transformative societal change.

With respect to the qualities of co-production identified by Chambers et al. (2021), our findings emphasise capacity development and networks, process quality and learning and the uptake of policy and management practices. In addition, interviewees commented on outputs related to institution building, trust building, social equitability and empowerment that echoes the findings of Kuchenmüller et al. (2022) on the domains of institutionalisation.

We argue that the distinction between direct instrumental use of co-production based on immediate needs and the long-term building of a stronger base for making transformative change is relevant in developing the institutional domains for knowledge co-production. A needs-based co-production of knowledge can be significantly advanced by institutionalising funding mechanisms and processes for the identification of knowledge needs. The current structure of the SRC largely achieves this. The other domains of institutionalisation such as specific rules and standardisation of approaches are not critical although they may hinder sudden politically motivated deinstitutionalisation. Transformative approaches to production of knowledge and solutions clearly need institutionalisation beyond funding, and the identification of needs to be seen as legitimate and to ensure uptake of results. Developing governance by introducing structures such as science panels with a formal role in the policy making is one way of taking the institutionalisation further.

### Challenges and opportunities for institutionalisation

Generic challenges in the institutionalisation of co-production include issues of effectiveness, accountability, inclusiveness, and commodification (e.g. Joshi and Moore 2004; Steen et al. 2018). Our results suggest that the perceived role of the co-production of knowledge affects both the opportunities for and paths towards institutionalisation.

*In needs-based approaches,* the power and leadership are in the hands of the knowledge users as they have specified what kind of knowledge should be delivered. They design the governance routines, specify the support and provide resources. The research community is expected to engage in collective action and commit their skills to serve the specified needs. In the best of cases, this can build culture of openness and dialogue. Less positive scenario of the institutionalisation is that it reduces the co-production to an activity in which the powerful set rules that stifle creativity and true dialogue.

The institutionalisation process depends on political power and agency (Cooper et al. 2008). Resources that are based on discretionary decisions can disappear at the stroke of a pen and reverse the institutionalisation. This was forcefully demonstrated by the new conservative government of Finland that took office in June 2023 (Government of Finland 2023) when it axed the funding for the Government’s analysis, assessment and research activities (VNTEAS),^2^ without even consulting the ministerial civil servants. The Government’s quest to cut costs and to rule by solutions it has specified in advance meant that the VNTEAS was seen as redundant. There were also critical voices raised against the SRC funding, but because the SRC has been institutionalised by law, cutting the resources of SRC would have been politically difficult. Interestingly, the VNTEAS was partly re-established in 2025 due to strong demands from key ministries. This suggests that the co-production process has reached some institutionalisation in the domain of culture in the Finnish science-policy interface.

*Transformative approaches to co-production,* as shown in our results, build on accepting complexity and uncertainty and a search for potentially radical change relative to status quo. This gives, as in the case of the SRC projects, the research community greater freedom in setting the agenda. However, SRC projects have, based on this, also been criticised by politicians that the researchers have tried to advance political rather than scientific goals. This further highlights the points raised by Cooper et al. (2008) that agency and power are essential to consider in analyses of institutionalisation.

At a practical level, our results show that the institutionalisation of co-production has progressed in the activities funded by SRC in all the domains identified by Kuchenmüller et al. (2022), but the pace and degree vary. The general governance is the most prominent domain of institutionalisation when it comes to funding that can be used for co-production. Within the sectors that we have examined the sector specific governance does not include rules on the co-production of knowledge, but a general demand to use participatory approaches in, for example, land-use planning, can be seen to establish a favourable governance environment for the further institutionalisation of knowledge co-production. This is, however, still far from achieving fully standardised and routinised processes. In the cases we studied partnerships and ad hoc collective action were achieved, but the long-term co-creation of knowledge outside the framework and support of the SRC remained challenging. The same conclusion has been reached by Tudose et al. (2023). It would, however, be too simplistic to state that this challenge could be overcome by dedicating more resources for co-production. There are, as pointed out by Kuchenmüller et al. (2022), interactions between the domains, which implies that also power and agency come into play (Cooper et al. 2008). Thus, resources are likely to be inefficiently used, if there is, for example, a lack of long-term commitment and leadership or strong political opposition. Our interviews also highlighted complexity and the need for learning concepts to deal with it as obstacles to co-production. Ruoslahti (2020) specifically examined the role of complexity in the co-creation of knowledge for innovation and found that it is essential for co-creators to tackle complexity systematically to “create new order”. This is particularly relevant for transformative co-production of knowledge. Thus, to become institutionalised, transformative co-production needs to be supported by capacity building and the building of trust across the science-policy interface. Without trust, it is difficult if not impossible to address complexity, uncertainty and contested problem definitions.

Our findings suggests that to build trust in co-production of knowledge, researchers need to learn to design and carry through effective, efficient, motivating and meaningful co-production processes. The emerging trust in relationships between the knowledge producers and users was raised as an important output for authentic knowledge co-production, as research from other fields also suggests (Boyle et al. 2023; Jakoet-Salie and Ramolobe 2023). This can be supported with training and guiding provided in the SRC projects and other similar funding that includes the co-production as a criterion for funding. Moreover, the participation in such projects broadens the networks and enhances opportunities for low threshold interaction with relevant stakeholders to continue discussions on the nature of the problems, the required knowledge and co-production and the potential solutions and knowledge gaps to evaluate them. Such capacity and network building provide key opportunities for further institutionalisation of knowledge co-production. However, recent political developments in many parts of the world demonstrate that populist movements can deliberately erode trust in research and thereby strive to destabilise the institutionalisation of co-production of knowledge.

There are other activities and efforts beyond the SRC that can be seen to develop a culture of co-production of knowledge. The heterogeneity of these initiatives and the fluidity of the formalisation of co-production may be fruitful in terms of outputs since the different options are tested and new innovative approaches may emerge (Kano and Hanashi 2021).

The creation of legally mandated expert panels is a strong form of institutionalisation of research input in policy development. When combined with a funding instrument such as the SRC, science panels can become important fora for co-creating sustainability transitions/ transformations. Empowerment of groups that have often been marginalised can also be achieved through specific bodies supporting co-production. For example, in the revision of the Finnish Climate Act (423/2022, Sect. 21) a dedicated Sámi Climate Council was established tasked with bringing the knowledge base and perspectives of the Sámi people into the climate policy processes.

### The need to recognise context in institutionalising processes of co-production of knowledge

Our results demonstrate that co-production often occurs in dialogues between researchers and civil servants that have in advance specified the knowledge needed. In many projects, the co-production process nevertheless develops ad hoc and depends greatly on the context and persons involved. There is thus a need to clarify expectations on co-production (Brandsen et al. 2018).

In co-production of knowledge, the domains of institutionalisation differ from sector to sector; for example, our interviews suggested that actors in the forestry sector perceive that co-production and partnerships have been part of the culture. However, our results also showed that such a culture may be seen to exclude researchers and actors that would be essential in co-producing of knowledge for sustainability transitions. For example, the forestry sector currently needs to consider not only traditional forestry values but also biodiversity and recreation and the rights of indigenous groups. The legacy effect of past activities potentially affects inclusiveness, equitability and diversity if the co-produced knowledge is perceived to be heavily influenced by a small active group (see also Steen et al. 2018, Gardiner et al. 2023).

It is relevant to recognise the differences between researchers’ and stakeholders’ perceptions of knowledge co-production. Stakeholders need to know what they are buying into when they are asked to institutionalise co-production processes, and researchers need to be clear about what they offer when they claim to deliver co-produced knowledge. A research funding mechanism, such as the SRC, that emphasises and supports explorative testing of knowledge co-production, can be a step towards more advanced institutionalisation in all domains. Further research is needed on ways of extending the impact of transdisciplinary sustainability research beyond the conventional boundaries of research projects (see, for example, Scaini et al. 2024).

It is important to note that the institutionalisation of knowledge co-production raises expectations among stakeholders. Thus, the creation of national panels that deal with such topics as climate change, biodiversity, natural resources and sustainable development may generate high hopes that such panels will deliver normative knowledge for transformative change. For example, in its statement to the government on the draft decree establishing a Nature Panel, the Finnish Association for Nature Conservation emphasised that “the Panel is important for stopping biodiversity loss” (Veistola 2023). However, other stakeholders, such as the forest owners, are likely to expect support and justification for modest incremental policy change. If the tensions between the views are left unresolved, they may lead to frustration and even the rejection of co-production. Unacknowledged differences in knowledge sensemaking can contribute to the “dark side” of co-production, i.e. the masking of potential pitfalls by the optimistically understood term (Steen et al. 2018). For example, if one participant group assumes that it can “order” and legitimise specific results through the co-production, whereas another expects open-ended deliberation towards consensus, the co-production may end up in reinforcing biases or depend on power structures that increase distrust between the actors. This may also discredit the co-created knowledge and its institutional base in the eyes of stakeholders not participating in the process.

A shift from exploratory testing to advanced institutionalisation is thus not without challenges. An open public dialogue that critically examines and enables the different approaches to be heard and understood is likely to help in clarifying expectations on co-production and its institutionalisation.

## Conclusions

The institutionalisation of knowledge co-production in sustainability research has potential for more impactful science-policy interactions. Our study shows that a funding instrument for research that is based on co-production leads to considerable diversity in implementation: practically all projects of our case study placed a strong emphasis on interaction, but few extended all the way to the reciprocal transformation of societal output, which is often considered the goal of sustainability research.

The observed differences in sensemaking of knowledge co-production have implications for how knowledge co-production can be institutionalised. Funding instruments, such as that of the Strategic Research Council in Finland, can create a demand for novel research approaches and develop the skills of researchers to engage in science-policy interactions by offering resources for explorative activities. In need-based co-production, this may be sufficient. Advanced institutionalisation of transformative co-production requires that attention is paid also to other domains such as governance, the standardisation and routinisation of processes, partnerships, leadership, culture and commitment in particular on the part of the users of co-produced knowledge. Transformative change is likely to come about through combinations of need-based co-produced knowledge for incremental policy change and the institutionalisation of knowledge co-production that fundamentally challenges business as usual.

## Supplementary Information

Below is the link to the electronic supplementary material.

Supplementary file1 (PDF 564 KB)

## Data Availability

The anonymised data that support the findings of this study and that are not shared in the Supplementary materials are available from the authors upon reasonable request.

## Footnotes

1 A distinction has been made between transdisciplinarity that tends to search for a “unity of knowledge” (“Mode 1”) and more practical transdisciplinarity aiming for participatory problem-solving approaches that are applied to tangible real-world problems (“Mode 2”) (Rigolot 2020). Here, we focus on the latter, and we use the term co-production as it is easy to conceptualise also for those who do not aim at “deeper” transdisciplinarity. This does not exclude the consideration of a more theoretical approach, and where relevant we specifically refer to transdisciplinarity to emphasise efforts to go beyond the practical solutions towards unity of knowledge.

2 https://valtioneuvosto.fi/en/governments-analysis-assessment-and-research-activities [visited February 12th 2025].
