# Supplementary material for: The emerging institutionalisation of knowledge co-production in sustainability research
Source: Ambio. 2025 Mar 18;54(8):1372–85. doi: 10.1007/s13280-025-02161-5 (PMC12214086; doi:10.1007/s13280-025-02161-5)
Supplement: Supplementary file 1 — Supplementary file1 (PDF 564 KB) [file 13280_2025_2161_MOESM1_ESM.pdf]

**Ambio**

Supplementary Information

*This supplementary information has not been peer reviewed.*

Title: **The Emerging Institutionalisation of Knowledge Co-Production in Sustainability Research**

## ANNEX S1 — interview guide

| # | Questions and themes |
|---|---|
| **Theme:** | **Introduction: consent, recording, basic information on the research and about the case** |
| **Theme:** | **Background information of participants** |
| **1** | What is you role in SRC projects and how is it related to SRC activities? |
| **1.1** | Which SRC projects are relevant for your work and what is your role? |
| **2** | What is your experience with the societal interaction methods used in the SRC projects? |
| **Theme:** | **Knowledge co-production experience and perceptions** |
| **3** | Have you participated in knowledge co-production in the SRC projects at different phases of the project? |
| **4** | How would you describe the knowledge co-production process in the SRC projects with regard to its purpose, impacts, agency, power relations? |
| **5** | In your opinion, how has knowledge co-production succeeded in the SRC projects that you are familiar with? |
| **5.1** | *Follow-up question:* What would you describe as your/your organisation's role in its success? |
| **5.2** | *Follow-up question:* Has the co-produced knowledge been used in decision-making? What factors influenced it being considered during decision-making? |
| **5.3** | *Follow-up question:* Have the SRC projects worked as a platform for knowledge exchange and/or for fostering mutual understanding? |
| **6** | What would you perceive as the main challenges for the success of knowledge co-production? |
| **6.1** | *Follow-up question:* Have you identified specific challenges related to acceptability, funding, other sources of knowledge co-production, sensitivity of the research topics, communication? |
| **7** | Does the explicit use of 'knowledge co-production' in SRC projects have any specific influences? |
| **7.1** | *Follow-up question:* Have you identified ways in which it influences the results, implementation of the results, credibility, participation? |
| **Theme:** | **Institutionalisation of knowledge co-production** |
| **8** | Can you identify any unexpected impacts of the SRC projects or the SRC instrument? |
| **9** | When you as a knowledge user need new knowledge, how do you perceive your options for gaining it? |
| **10** | What role does funding play in the implementation and impacts of co-production? |
| **Theme:** | **Perception of knowledge co-production** |
| **13** | Has the knowledge co-production process in SRC projects changed your understanding of societally impactful research? |
| **14** | What will be the role of knowledge co-production in the future? |
| **15** | Would you like to add something before we finish? |

Table S1. Interview guide (a translated summary of the original Finnish interview guide)

**ANNEX S2 — Interviews**

| Interview code | Participant information |
|---|---|
| 1. | Project advisory board (ministry) |
| 2. | Expert in SRC funding development (ministry) |
| 3. | Municipal-level decision maker |
| 4. | Adv. board (ministry) |
| 5. | Association of Finnish Municipalities |
| 6. | Finnish Climate Panel (ministry) |
| 7. | Adv. board (ministry) |
| 8. | Stakeholder (ministry) |
| 9. | Expert in SRC funding/research development (ministry) |
| 10. | Adv. board (Env. org.) |
| 11. | Adv. board (Nat. innovation fund) |
| 12. | Head of Research (ministry) |
| 13. | SRC coordination |
| 14. | Expert in SRC funding/research development (ministry) |
| 15. | Adv. board/sectoral partner (Energy sector) |
| 16. | Adv. board/ municipal decision-making |

Approximately half of all persons invited to the interviews agreed to participate in them. The snowballing method was applied in the invitations and interviews, i.e. interviewees were asked to identify potential additional persons of interest. Half of the final list of interviewees are snowball sampled.

Table S2. Interview information.

**ANNEX S3 — Framing analysis**

From the modes presented by Chambers et al. (2021), two of the themes, purpose and pathways, were corresponding to the themes of the study. 'Purpose' describes why participants want to co-produce the knowledge and 'pathways' how the impacts are catalysed (*ibid*.). To operationalise the framework, we adopted the categories of co-production purpose and impact pathways used by Chambers et al. (2021) and linked them with the levels of institutionalisation. In exploring the level of institutionalisation, we emphasised the domains of governance, routinisation, commitment, partnerships and resources. The purpose of co-production is defined in relation to how efforts at (re)defining and solving the societal problem(s) in question are approached. The impacts of co-production are assessed by examining such outcomes as knowledge production and transfer, capacity development and networks, process quality and learning, empowerment and social equitability, institution building, policy uptake and management practices, ecological outcomes and social outcomes and trust (Chambers et al., 2021). The outcomes also provide indications of the progress of institutionalisation especially in the domains of collective action and culture. In practice, we focus on outputs and intended outcomes rather than on achieved outcomes, as the latter are difficult or impossible to verify when assessing on-going processes. Concrete outputs, such as joint events, documents, and contributions to regulatory processes, are considered here as a precondition for future outcomes. Our analysis examines, in particular, those outputs and intended outcomes that are likely to encourage both users of research results and researchers to engage in co-production of knowledge, thereby supporting its further institutionalisation.

Initial coding consisted of the seven-tier scale for two different approaches on the co-production process on each of the two themes, resulting in total of 28 *a priori* codes derived from the framework of Chambers et al. (2021). JK and IH made the preliminary coding; thereafter the analysis was conducted with all authors involved. Empirical category for the emerging institutionalisation was identified after first round of coding. Based on abductive logic, the analysis proceeded with returning to theory. Codes were merged into six categories constituting of a matrix with three columns presenting two extreme ends and one intermediate category of the degree of formalisation of co-production (cf. Joshi & Moore, 2004), and the seven-tier scale for classifying the purpose and pathway was modified into a four rows presenting the categories of framing (Entman, 1993) and practices of co-production (Chambers et al., 2021). As Gibson and Brown (2009) describe, *a priori* codes present the more general idea of the topic researched and empirical codes and categories reveal what is less explored on the phenomena.

| | **Explorative institutionalisation** | **Emerging institutionalisation** | **Established institutionalisation** |
|---|---|---|---|
| **Purpose of knowledge co-production in relation to solving problems (*cf.* Chambers et al., 2021):** | Not focused on solutions (the approach does not focus on solutions, e.g. scenario building, workshops to learn about stakeholder perceptions); solutions may emerge from the process | Different solutions may be generated/clarified/narrowed down/negotiated during the process | Focused on solutions that are suggested, fixed early on in the process; possibly revise approaches to pursue them during the process |
| Respondents (interview)[1] | 3. | 13. | 1., 5., 6., 12. |
| Number of projects mentioned regarding the output/projects that the interviewees were involved with | 1 | 1 | 11 |
| Number of projects related to the survey responses | 1 | - | 1 |

[1] Numbers refer to the list of respondents (see Annex B).

| | | | |
|---|---|---|---|
| **Purpose of knowledge co-production in relation to reframing research problems (*cf.* Chambers et al., 2021):** | No process in place for questioning initial problem (or solution) framework | Providing space for reframing when necessary | Active reframing of the problem and the solutions together with stakeholder participation |
| Respondents (interview) | 2., 10. | 9., 13. | 5., 15., 16. |
| Number of projects (interview) | 5 | several | 5 |
| Number of projects (survey) | - | 2 | 1 |
| **Impact pathways in relation to producing knowledge (*cf.* Chambers et al., 2021):** | The value of knowledge production is purely in the value of new knowledge itself and/or in its theoretical implications for change; impacts cannot be prepared for | Producing and/or transferring *usable* knowledge/knowledge for a certain purpose/common interest can influence or even catalyse an impact | Knowledge production and transfer are the key catalysts for the impact, i.e. the impact is not possible without new knowledge and/or its transfer |
| Respondents (interview) | 1., 3., 6., 13., 15. | 5.-8., 10., 14.-16. | 4., 8., 11., 13. |
| Number of projects (interview) | 8 | 15 | Several |
| Number of projects (survey) | 1 | 1 | 1 |
| **Impact pathways in relation to relating together/interactions between the researchers and the decision makers (*cf.* Chambers et al., 2021):** | Intensive interaction (bi-directional, iterative, often, systematic, started early on, doing together, shared interests) | In between (intensive in some parts/phases, loose in others; not realising the full potential of knowledge co-production) | Loose interaction (uni-directional, instrumental, randomly/rarely done, general level; e.g. focus on the advisory board activities, general-level policy briefs and providing of other information) |
| Respondents (interview) | 6., 9., 13., 14., 16. | 1., 2., 13. | 2., 4., 6., 13., 16. |
| Number of projects (interview) | 10 | several | 13 |
| Number of projects (survey) | 7 | 3 | - |

Table S3. The framing analysis coding matrix.

## References


Chambers, J.M., Wyborn, C., Ryan, M.E., Reid, R.S., Riechers, M., Serban, A., Bennett, N.J., Cvitanovic, C., et al., 2021. Six modes of co-production for sustainability. *Nature Sustainability* 4.11: 983-996. https://doi.org/10.1038/s41893-021-00755-x

Gibson, W.J. and Brown, A., 2009. *Working with Qualitative Data in Chapter 8: Identifying Themes, Codes and Hypotheses*. SAGE Publications, Ltd. //doi.org/10.4135/9780857029041

Joshi, A. and Moore, M., 2004. Institutionalised co-production: unorthodox public service delivery in challenging environments. *Journal of Development Studies*, *40*(4), 31-49. https://doi.org/10.1080/00220380410001673184

**Annex S4 - Survey response**

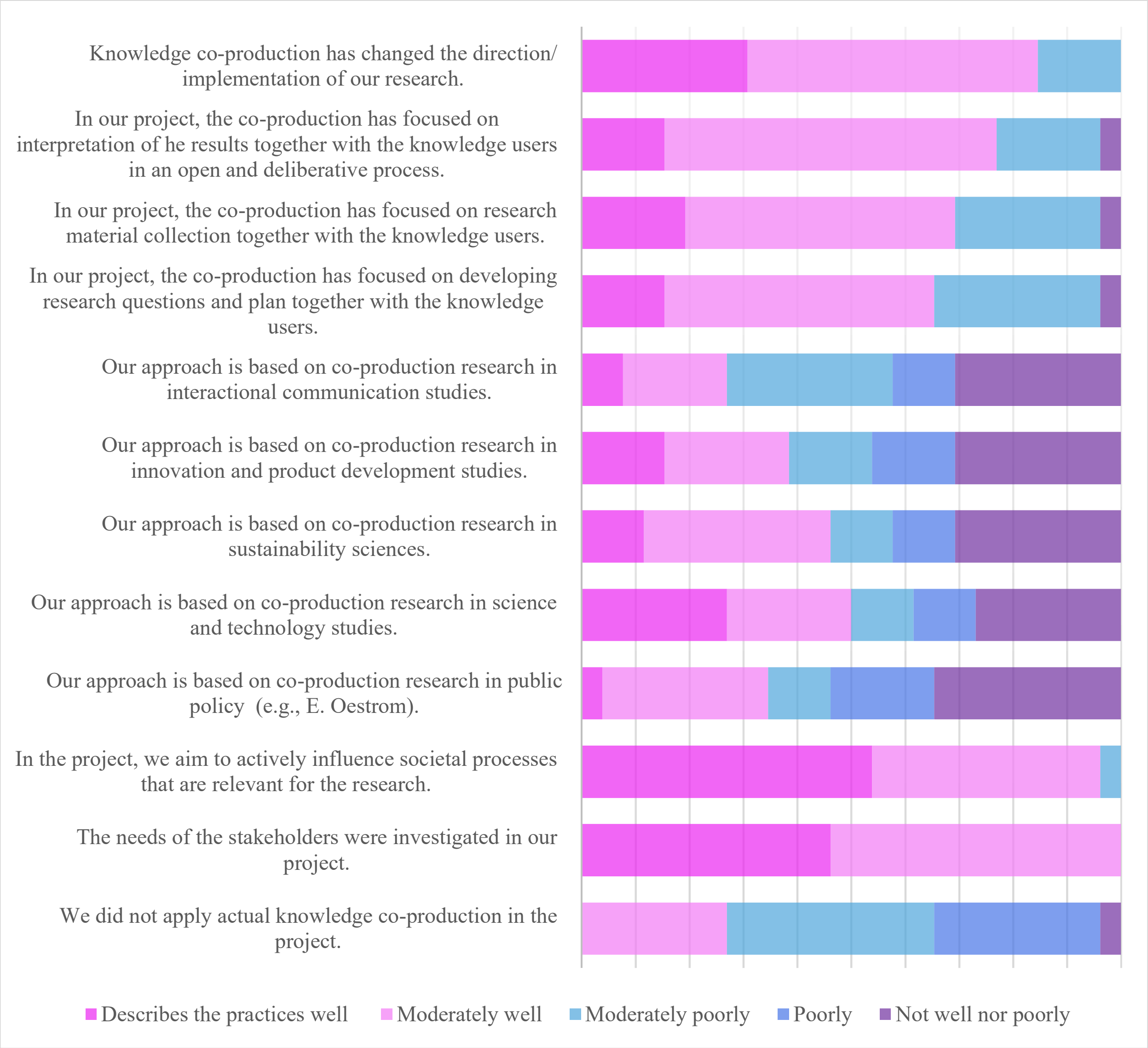


Figure S4.1. Distribution of survey responses regarding knowledge co-production practices: N = 25 (projects).

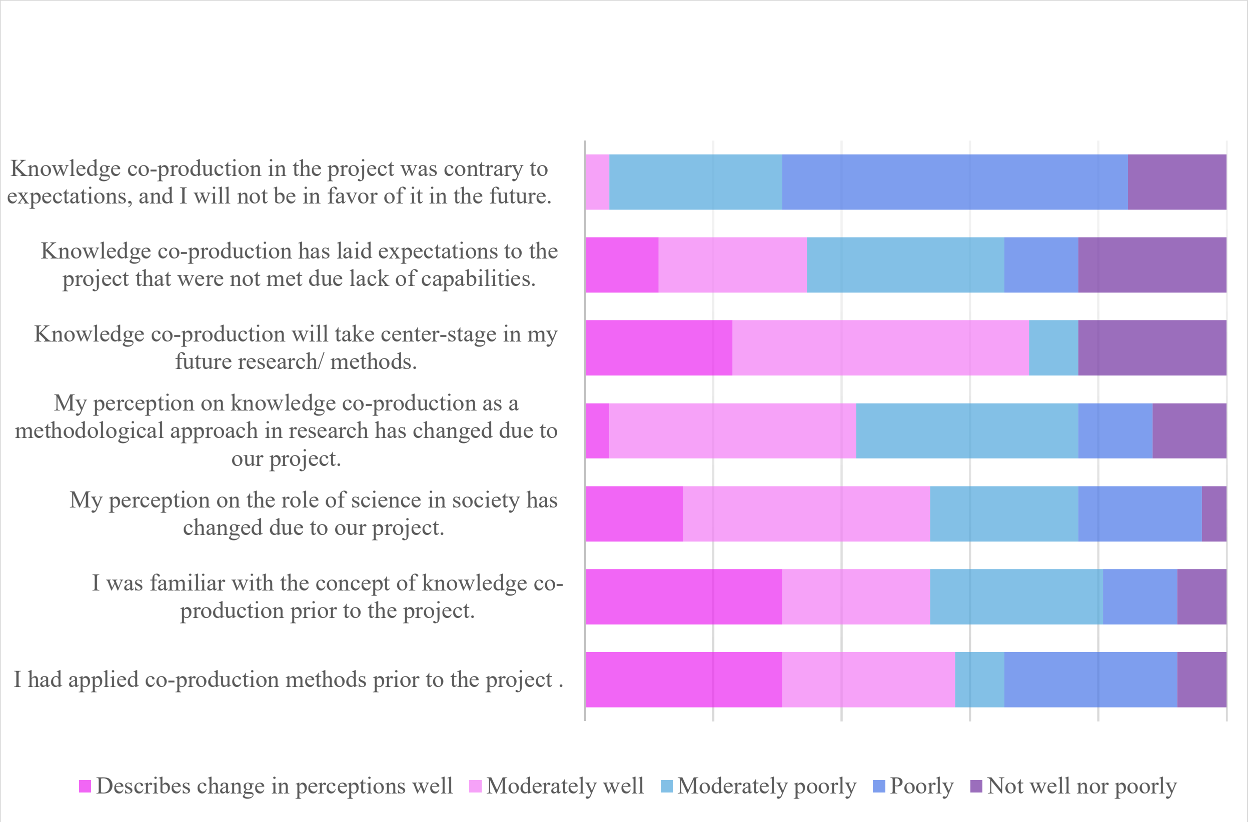


Figure S4.2. Distribution of survey responses regarding perceptions of the knowledge co-production process: N = 25 (projects).

**ANNEX S5 – The SRC case description**

The SRC was created by a political decision in the early 2010s that cut a significant portion of budgetary funding for government research institutes to establish a new funding instrument that would deliver long-term research supporting strategic planning and decision making. It marked the beginning of the institutionalisation of a new type of nationally targeted, transdisciplinary research in Finland. The aim was to fund research that would flexibly address grand societal challenges (Government Bill HE 25/2014 vp).

The societal problems that the SRC projects are expected to address are specified at a general level for each funding call as a 'theme'. The themes that the SRC funds are developed in an interactive manner involving both researchers and knowledge users, before ultimately being approved by the government (Act on the Academy of Finland 2009/922). The researchers competing for funding are given considerable freedom in identifying the specific topics and problems and in proposing particular ways to address them. The projects are selected for funding based on assessments and recommendations by independent panels, with final decisions made by the SRC.

In practice, the SRC annually funds two or three thematically unified programmes with 3–7 multidisciplinary consortiums, each receiving yearly funding of approximately 1 MEUR for up to six years. The first SRC programmes were launched in 2015, and new programmes have been launched yearly ever since. The programmes have covered societal challenges broadly, ranging from carbon neutrality and natural resources to technological transitions and social equity. Two programmes have included consortia that focused on environmental issues from the perspective of participatory decision-making. Of the ongoing thirteen programmes that began in 2018 or later, six programmes and two individual consortia in a governance programme have focused on environmental topics.

**References**

Act on the Academy of Finland 2009/922. Retrieved on February 2024 from
https://www.finlex.fi/fi/laki/ajantasa/2009/20090922
